# Investigating Rubidium Density and Temperature Distributions in a High-Throughput ^129^Xe-Rb Spin-Exchange Optical Pumping Polarizer

**DOI:** 10.3390/molecules28010011

**Published:** 2022-12-20

**Authors:** James E. Ball, Jim M. Wild, Graham Norquay

**Affiliations:** POLARIS, Department of Infection, Immunity & Cardiovascular Disease, The University of Sheffield, Sheffield S10 2TA, UK

**Keywords:** NMR, MRI, hyperpolarization, spin-exchange optical pumping, xenon-129, rubidium, spectroscopy, atomic, absorption

## Abstract

Accurate knowledge of the rubidium (Rb) vapor density, [Rb], is necessary to correctly model the spin dynamics of ${}^{129}$Xe-Rb spin-exchange optical pumping (SEOP). Here we present a systematic evaluation of [Rb] within a high-throughput ${}^{129}$Xe-Rb hyperpolarizer during continuous-flow SEOP. Near-infrared ($5^{2}S_{1/2}\to 5^{2}P_{1/2}$ (D${}_{1}$)/5${}^{2}P_{3/2}$ (D${}_{2}$)) and violet ($5^{2}S_{1/2}\to 6^{2}P_{1/2}$/6${}^{2}P_{3/2}$) atomic absorption spectroscopy was used to measure [Rb] within 3.5 L cylindrical SEOP cells containing different spatial distributions and amounts of Rb metal. We were able to quantify deviation from the Beer-Lambert law at high optical depth for D${}_{2}$ and 6${}^{2}P_{3/2}$ absorption by comparison with measurements of the D${}_{1}$ and 6${}^{2}P_{1/2}$ absorption lines, respectively. D${}_{2}$ absorption deviates from the Beer-Lambert law at ${\left[\mathrm{Rb}\right]}_{D_{2}}>4\times 10^{17}$ m${}^{-3}$ whilst 5${}^{2}$S${}_{1/2}\to 6^{2}P_{3/2}$ absorption deviates from the Beer-Lambert law at ${\left[\mathrm{Rb}\right]}_{6P_{3/2}}>\left(4.16\pm 0.01\right)\times 10^{19}$ m${}^{-3}$. The measured [Rb] was used to estimate a ${}^{129}$Xe-Rb spin exchange cross section of $\gamma^{\prime }=\left(1.2\pm 0.1\right)\times 10^{-21}$ m${}^{3}$ s${}^{-1}$, consistent with spin-exchange cross sections from the literature. Significant [Rb] heterogeneity was observed in a SEOP cell containing 1 g of Rb localized at the back of the cell. While [Rb] homogeneity was improved for a greater surface area of the Rb source distribution in the cell, or by using a Rb presaturator, the measured [Rb] was consistently lower than that predicted by saturation Rb vapor density curves. Efforts to optimize [Rb] and thermal management within spin polarizer systems are necessary to maximize potential future enhancements of this technology.

## 1. Introduction

Hyperpolarized ${}^{129}$Xe magnetic resonance imaging (MRI) is used for pulmonary research and clinical diagnostic imaging, providing insight into many lung conditions including chronic obstructive pulmonary disease [1], asthma [2], cystic fibrosis [3] and COVID-19 [4,5]. In addition, the high solubility of xenon in blood and tissues enables MR imaging of xenon in well perfused organs such as the brain [6], kidneys [7] and heart [8], offering a unique diagnostic tool to quantify physiological parameters associated with pathologies in organs beyond the lungs.

To generate "hyperpolarized" ${}^{129}$Xe gas samples, the technique ${}^{129}$Xe-Rb spin-exchange optical pumping (SEOP) is used to elevate the nuclear spin polarization of ${}^{129}$Xe ($P_{\mathrm{Xe}}$) from its thermal equilibrium value of $∼1\times 10^{-6}$ on clinical 1.5T MRI scanners up to values >0.1, resulting in five orders of magnitude MR signal enhancement. During ${}^{129}$Xe-Rb SEOP, spin angular momentum is transferred from a beam of circularly polarized photons to ${}^{129}$Xe nuclei via collisional Fermi-contact hyperfine interactions with optically polarized Rb valence electrons [9], leading to a build up in ${}^{129}$Xe nuclear spin polarization. Practically, ${}^{129}$Xe-Rb SEOP is performed using one of two methods: "batch-mode" (stopped-flow) [10,11] and "continuous-flow mode" [12,13,14,15]. Batch-mode SEOP generally involves Xe-rich gas mixtures in combination with low Rb vapor density, [Rb], in order to maintain high Rb polarization in the presence of a high Rb-Xe electronic spin destruction cross section. The SEOP cell contains a fixed volume of gas which is held within the cell for the duration of ${}^{129}$Xe polarization build up. Once high $P_{\mathrm{Xe}}$ is reached, the hyperpolarized gas mixture is collected without the need for cryogenic separation. Continuous-flow mode SEOP involves passing a lean Xe (typically 1–3%Xe) gas mixture through the SEOP cell and cryogenically separating it from buffer gases within liquid-N${}_{2}$-submerged glassware (cryo-trap). Given the relatively short residency time (of the order 1 min) of Xe in the cell during flow, high [Rb] is required to increase the ${}^{129}$Xe-Rb spin-exchange rate to ensure sufficiently high $P_{\mathrm{Xe}}$ values are reached before the gas exits the cell. High gas flow rates are important to reduce polarization losses of frozen Xe within the cryo-trap, and to maximize Xe production rates in a clinical setting where on-demand doses are required.

Within continuous-flow SEOP setups, large and small volume SEOP cells have been implemented. Historically, small SEOP cells were used with high gas pressures to (i) increase the Xe residency time and (ii) pressure-broaden the D${}_{1}$ linewidth to improve absorption efficiency for the ∼2–3 nm linewidth laser diodes that were available at the time [12]. The development of high power laser diodes (>100 W) with <0.3 nm linewidths has made it possible to maintain high absorption efficiency at lower gas pressures. At lower gas pressures, three-body van der Waals (vdW) molecules contribute to spin-exchange [16], increasing the ${}^{129}$Xe-Rb spin exchange cross section and resulting in faster polarization build-up rates. To date, large volume cells (>1 L) have been shown to be the most effective on continuous-flow polarizers for maximising light absorption and achieving rapid volume production of highly polarized ${}^{129}$Xe [13,14,15].

While near-unity ${}^{129}$Xe polarization has been achieved in a batch-mode SEOP system [11], continuous-flow systems suffer from under performance based on current theoretical frameworks [14,15,17,18,19]. Kelley and Branca [20] addressed discrepancies with reported ${}^{129}$Xe-Rb spin-exchange rates, arriving at a closed-form expression similar to that of Walker and Larsen [21], and suggested that variations in calculated [Rb] when measuring binary and vdW spin-exchange cross sections in previous studies contributed significantly to the widely reported discrepancies between theoretical and experimental polarizer performance. Indeed, Kelley and Branca [20] measured [Rb] much lower than saturation [22] in a small-cell continuous-flow SEOP setup which, unless accounted for, would lead to significant underestimations in measured ${}^{129}$Xe-Rb spin-exchange cross sections. In large SEOP cell setups, indirect observation of lower than expected [Rb] has been made by Plummer et al. [23]. To our knowledge, [Rb] and its distribution within the SEOP cell has not yet been measured directly within large SEOP cell polarizers that implement high power (>100 W), spectral-narrowed (<0.3 nm) laser diodes.

Typically for our setup, the required [Rb] in the SEOP cell is created by placing a droplet of Rb within the heated and illuminated main body of the cell. Over time, evaporation of Rb from the Rb droplet leads to a saturated Rb vapor density, ${\left[\mathrm{Rb}\right]}_{\mathrm{sat}}$. Unlike batch mode production, where the gas is sealed within the SEOP cell during polarization build up, in continuous-flow setups, gas flow may disturb [Rb] in the SEOP cell. If Rb vapor is displaced by the gas flow faster than it can be replaced by Rb evaporation from the Rb sources, then [Rb] will be lower than ${\left[\mathrm{Rb}\right]}_{\mathrm{sat}}$. Also, lower-than-expected [Rb] in SEOP cells have been observed where the surface area of the Rb coating is low [24]. The surface area of Rb in the main body of the cell could be increased, however this may increase susceptibility to Rb runaway [25]. This is where continuous coupling of laser heating and Rb evaporation is established resulting in a highly opaque region within the SEOP cell, which can lead to dark Rb (i.e low Rb polarization due to low optical pumping rate) regions of the cell. In continuous-flow SEOP with large SEOP cells, this is particularly challenging compared to small SEOP cell setups as thermal management demands are higher due to the larger SEOP cell volume, as well as the higher degree of laser heating, owing to the high laser powers used and the higher proportion of laser absorption. To improve [Rb] levels, and mitigate Rb runaway effects, Rb presaturation regions have been implemented on other polarizer systems [13,14,23,26]. This is an upstream section of the SEOP cell where the Rb sources are placed and heated. The Rb presatuation region is not illuminated by the pumping laser, thus decoupling laser heating from Rb evaporation. However, lower than saturation Rb densities have still been observed in these setups [23,26].

Atomic absorption spectroscopy can be used to directly measure Rb and other alkali metal vapor densities in SEOP cells [27,28,29]. Consideration of the optical thickness of the atomic transition probed is important as deviation from the Beer-Lambert law can occur at high optical thickness, resulting in reduced accuracy of determined [Rb] when the Beer-Lambert law is assumed. However, reduced optical thickness typically also comes at the expensive of lower signal-to-noise ratio (SNR), making large ranges of [Rb] difficult to measure using only one absorption line. Therefore, simultaneously probing multiple transitions can extend the range of measurable [Rb] for a setup. Comparison of [Rb] determined for each transition allows reduced measurement accuracy as a result of breakdown in the Beer-Lambert law for high absorbances to be characterised.

The motivation for this work was to improve the understanding of Rb vapor dynamics in a clinical-scale continuous-flow SEOP hyperpolarizer. We investigated in-cell [Rb] heterogeneity using near-infrared (NIR) and violet atomic absorption spectroscopy on cells with differing spatial distributions of Rb sources. We also measured cell temperature distribution to assess thermal management and its role in [Rb] distributions. We systematically characterized the validity of the Beer-Lambert law for each absorption line over a range of [Rb]. We used [Rb] measurements from atomic absorption spectroscopy to measure the ${}^{129}$Xe-Rb spin-exchange cross section and compared this to values derived from the literature.

## 2. Materials and Methods

### 2.1. SEOP Theory

The two-stage process of SEOP involves (i) the optical pumping of Rb valence electrons, leading to high Rb electronic polarization and (ii) spin-exchange between Rb valence electrons and ${}^{129}$Xe nuclei. [Rb] governs the spin dynamics during both stages of SEOP. The Rb polarization, $P_{\mathrm{Rb}}$, is described by

(1) $$P_{\mathrm{Rb}}=\frac{R}{R+\Gamma_{\mathrm{sd}}},$$

where *R* is the optical pumping rate and $\Gamma_{\mathrm{sd}}$ is the Rb electronic spin-destruction rate. *R* relates $P_{\mathrm{Rb}}$ to the rate at which circularly-polarized photons are absorbed per Rb atom, $\delta \Gamma =(1-P_{\mathrm{Rb}})R$, and has a value of $R=\beta P_{l}n_{p}/A$ at the SEOP cell incidence. $P_{l}$ is the laser power, *A* is the beam area and $n_{p}$ is the number of photons per Joule at the pump laser center wavelength, $\lambda_{l}$. β is the coefficient relating the photon flux ($P_{l}n_{p}/A$) to *R*, which for a Gaussian laser profile has previously been shown to be [30,31]

(2) $$\beta =\frac{2\sqrt{\pi \ln2}r_{e}f_{D_{1}}\lambda_{l}^{3}w^{\prime }\left(r,s\right)}{hc\Delta \lambda_{l}n_{p}},$$

where $\Delta \lambda_{l}$ is the pump laser linewidth. $w^{\prime }\left(r,s\right)$ is the real part of the complex overlap function, *w*, given by $w=w^{\prime }+iw^{\prime \prime }=e^{\left[\ln2{\left(r+is\right)}^{2}\right]}\mathrm{erfc}\left(\sqrt{\ln2}\left[r+is\right]\right)$. Here $s=2\left(\nu_{l}-\nu_{a}\right)/\Delta \nu_{l}$ is the relative detuning and $r=\Delta \nu_{a}/\Delta \nu_{l}$ is the relative atomic linewidth of the atomic absorption line to the laser spectral output. Attenuation of *R* along the SEOP cell length, *z*, can be described by a non-linear differential equation [32]

(3) $$\frac{dR}{dz}=-R\beta \left[\mathrm{Rb}\right]\left(1-\frac{R}{R+\Gamma_{\mathrm{sd}}}\right).$$

The Rb-${}^{129}$Xe spin-exchange rate, $\gamma_{\mathrm{se}}=\gamma_{\mathrm{se}}^{\mathrm{bc}}+\gamma_{\mathrm{se}}^{\mathrm{vdW}}$, has contributions from binary collisions and the formation and break-up of RbXe vdW molecules. $\gamma_{\mathrm{se}}^{\mathrm{bc}}={\langle \sigma \nu \rangle }_{\mathrm{se}}\left[\mathrm{Rb}\right]$, where ${\langle \sigma \nu \rangle }_{\mathrm{se}}$ is the binary ${}^{129}$Xe-Rb spin-exchange cross section. The contribution to spin exchange from vdW interactions can be described by [21,30]

(4) $$\gamma_{\mathrm{se}}^{\mathrm{vdW}}=\frac{1}{2T_{K}}{\left(\frac{\phi }{x}\right)}^{2}\sum_{i}\eta_{i}\left(\frac{1+q_{i}{\left(\omega_{\mathrm{hf},i}\tau \right)}^{2}/{\left[I_{i}\right]}^{2}}{1+{\left(\omega_{\mathrm{hf},i}\tau \right)}^{2}}\right)=\gamma_{\mathrm{vdW}}^{\prime }\left[\mathrm{Rb}\right],$$

for $i\in {\{}^{85}$Rb, ${}^{87}$Rb}, where $\eta_{i}$ is the abundance of the Rb isotope *i*, [$I_{i}$]$=2I_{i}+1$ is the statistical weight of the Rb nuclear spin quantum number *I*, $\omega_{\mathrm{hf},i}$ is the hyperfine frequency, $q_{i}=1+\epsilon \left(I_{i},P_{\mathrm{Rb}}\right)$ is the paramagnetic coefficient for $P_{\mathrm{Rb}}$ within a spin-temperature distribution and $\gamma_{\mathrm{vdW}}^{\prime }$ represents the van der Waals ${}^{129}$Xe-Rb spin-exchange cross section. The *q* values for ${}^{87}$Rb (*I* = 3/2) and ${}^{85}$Rb (*I* = 5/2) isotopes are given by [30]

(5) $$1+\epsilon \left(\frac{3}{2},P_{\mathrm{Rb}}\right)=1+\frac{5+P_{\mathrm{Rb}}^{2}}{1+P_{\mathrm{Rb}}^{2}},$$

(6) $$1+\epsilon \left(\frac{5}{2},P_{\mathrm{Rb}}\right)=1+\frac{35+42P_{\mathrm{Rb}}^{2}+3P_{\mathrm{Rb}}^{4}}{3+10P_{\mathrm{Rb}}^{2}+3P_{\mathrm{Rb}}^{4}}.$$

$1/T_{K}=\left[\mathrm{Rb}\right]k/\tau$ is the RbXe molecular formation rate per Xe atom, *k*, is the molecular chemical equilibrium constant [33] and τ is the molecular lifetime, defined for any gas density composition ${\left[G\right]}_{i}$ by [15]

(7) $$\frac{1}{\tau }=\frac{\omega }{\phi }=\sum_{i}\frac{\gamma N}{\hbar }\frac{{\left[G\right]}_{i}}{{\left[G\right]}_{0,i}},$$

for i∈{Xe, N${}_{2}$, He}, where ${\left[G\right]}_{0,i}$ is defined as the characteristic third-body density for which the molecular break-up rate $\tau^{-1}$ is equal to the spin-rotation frequency, ω=γN/ℏ, of the Rb electron spin vector **S** about the rotational angular momentum vector **N** of the RbXe molecule. ϕ is the phase angle subtended by **S** within a molecular lifetime τ, γ is the coupling constant that determines the strength of the spin-rotation interaction γN·S [16] and *x* is the Breit-Rabi field parameter, which determines the fractions of Rb electronic *S* momentum that is transferred to rotational angular momentum *N* and to the ${}^{129}$Xe nuclear spin K=1/2. Spin-exchange parameter values are given in Table 1.

#### 2.1.1. ${}^{129}$Xe Polarization Build Up

${}^{129}$Xe polarization, $P_{\mathrm{Xe}}$, build up is described by the convection-diffusion partial differential equation

(8) $$\nabla \;\cdot \;\left(-D_{\mathrm{Xe}}\;\cdot \;\nabla P_{\mathrm{Xe}}\right)+v\;\cdot \;\nabla P_{\mathrm{Xe}}=\gamma_{\mathrm{se}}\;\cdot \;P_{\mathrm{Rb}}-\left(\gamma_{\mathrm{se}}+\Gamma^{\prime }\right)\;\cdot \;P_{\mathrm{Xe}},$$

where $D_{\mathrm{Xe}}$ is the Xe diffusion coefficient, v is the gas velocity and $\Gamma^{\prime }$ is the nuclear spin relaxation rate of ${}^{129}$Xe in the absence of Rb vapor. For continuous-flow SEOP at high gas flow rates, where gas flow dominates over Xe diffusion, and where gas flow is modelled as plug flow in the z-direction, Equation (8) reduces to one dimension

(9) $$P_{\mathrm{Xe}}=P_{\mathrm{Rb}}\;\cdot \;\frac{\gamma_{\mathrm{se}}}{\gamma_{\mathrm{se}}+\Gamma^{\prime }}\;\cdot \;\left(1-e^{-\left(\gamma_{\mathrm{se}}+\Gamma^{\prime }\right)t_{\mathrm{res}}}\right),$$

where $t_{\mathrm{res}}=z/v_{z}$ is the Xe residency time within the SEOP cell. The ${}^{129}$Xe polarization build up rate can be defined as $\gamma_{\mathrm{up}}=\gamma_{\mathrm{se}}+\Gamma^{\prime }$.

#### 2.1.2. Laser Heating

The dissipation of 10s of Watts of laser light absorbed during optical pumping through spin-relaxation contributes to the total thermal energy within the SEOP cell, and is given by [45]

(10) $$Q_{\mathrm{LH}}=h\nu_{l}\left[\mathrm{Rb}\right]R\frac{\Gamma_{\mathrm{sd}}}{R+\Gamma_{\mathrm{sd}}},$$

where *h* is Planck’s constant and $\nu_{l}$ is the laser frequency. Equation (10) shows that laser heating will occur in high areas of [Rb]. In addition, Equation (3) shows that high [Rb] will lead to greater gradients in the z-direction of *R*, and as a result, greater laser heating gradients across the cell.

### 2.2. SEOP Cell Rb Source Distributions and Rb Presaturation

**Figure 1:**
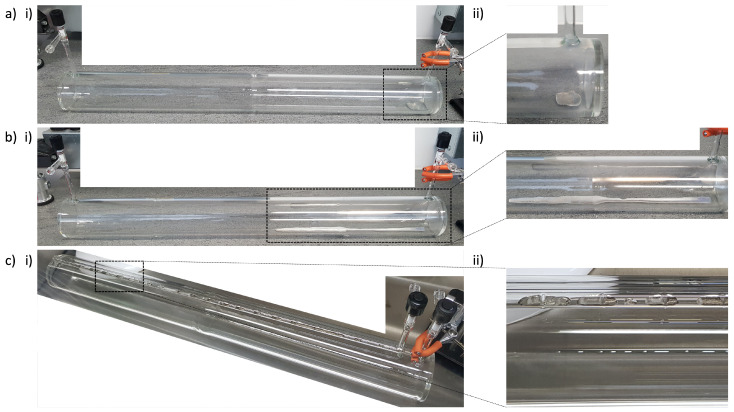
SEOP cells with different Rb source distributions; (**a**) 1 g of Rb placed at the back of the cell, with an estimated surface area ∼10 cm${}^{2}$; (**b**) 5 g of Rb placed in the cell, with an estimated surface area ∼64 cm${}^{2}$; (**c**) 2 g of Rb distributed along the presaturator, (**i**) showing the geometry of the cell and (**ii**) showing a close-up of the Rb distribution.

Cylindrical pyrex cells with internal diameter of $w_{\mathrm{cell}}=7.5$ cm and length of $L_{\mathrm{cell}}=79$ cm (external diameter, $w_{\mathrm{cell}}^{ex}=8.5$ cm and length, $L_{\mathrm{cell}}^{ex}=80$ cm) were used in this work, as in previous work [15]. All cells were cleaned with deionized water and isopropyl alcohol before drying at high temperature. Once dry, they were evacuated to an ultra-low pressure ($10^{-7}$ mbar) and placed in an argon glovebox for Rb filling. Three different Rb source distributions in the SEOP cells were produced, as shown in Figure 1. Two Rb distributions involved Rb placed inside the main body of the SEOP cell, whilst the third involved placing Rb only within a presaturation region upstream of the main body of the SEOP cell. The existing SEOP cell design was altered by extending the inlet to include a 72 cm presaturation region; this presaturator length was modelled previously to result in 87% Rb vapor saturation exiting the presaturator into the main cell body at high gas flow rates (Q=1.95 SLM) [46]. Heating tape with a maximum power output of 468 W was wrapped around a 53.5 cm section of the presaturation region in order to improve temperature control and provide a temperature gradient between the presaturator and the main body of the cell to increase Rb vapor diffusion into the main body of the cell during closed cell operation. A power supply of ∼21 W to the heating tape was used when using the presaturator.

### 2.3. Absorption Spectroscopy

**Figure 2:**
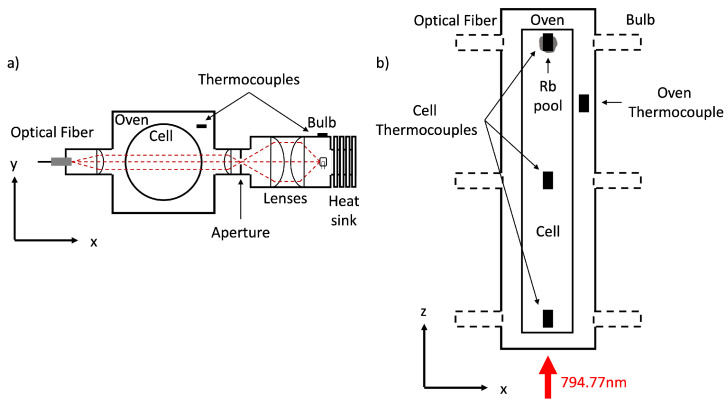
Experimental setup for atomic absorption spectroscopy. (**a**) Optical setup consisting of a halogen bulb as the broad spectral light source. A thermocouple is placed directly above the bulb on the housing to monitor bulb temperature and ensure the bulb has stabilized before measurements are taken. A series of plano-convex lenses (f = 40 mm, f = 60 mm, f = 30 mm) and aperture directs a parallel light beam onto the SEOP cell. A f = 75 mm plano-convex lens collects light and couples it to the optical fiber, which is connected to the spectrometer. (**b**) The optical setup was placed at 3 different positions along the length of the cell. At each position, cell temperature is measured by a thermocouple adhered to the top of the external cell surface. Air passed through a heating element, controlled by a thermocouple placed in the ambient oven space, regulates oven temperature. The oven has three inlets for heated air, to maximise heated air coverage and oven temperature homogeneity across the cell. To ensure oven performance is not compromised, the oven port and lens tube fit compactly and the remaining oven ports are filled with ceramic plugs lined with insulation foam. The optics run on rails connected to the polarizer chassis, ensuring precise optical alignment and fast re-positioning between oven ports. The direction of the optical pumping laser is shown, although this is switched off during atomic absorption spectroscopy acquisitions.

Rb vapor densities were measured using atomic absorption spectroscopy. This was performed using a similar setup to that described in previous work [29,47,48], as shown in Figure 2a. A 50 W halogen bulb provided a broadband spectral light source, which was directed onto the SEOP cell with a beam width of 21.1 mm, transverse to the pump laser beam direction. Light is then collected by a 75 mm plano-convex lens (Thorlabs) and coupled to an optical fiber (OceanInsight) and directed to the spectrometer (OceanInsight, model HR4000). Two spectrometers were used to observe Rb absorption lines in different frequency ranges, NIR and violet, at high resolution. The Rb D${}_{1}$ ($5S_{1/2}\to 5P_{1/2}$) and D${}_{2}$ ($5S_{1/2}\to 5P_{3/2}$) transitions, which lie within NIR, and the less attenuating $5S_{1/2}\to 6P_{1/2}$ and $5S_{1/2}\to 6P_{3/2}$ transitions, which lie within violet, were probed.

For high transmission, attenuation of the light can be described by the Beer-Lambert law as

(11) $$I\left(\nu \right)=I_{0}\left(\nu \right)e^{-[\mathrm{Rb}]l\sigma (\nu )},$$

where $I_{0}$ is the spectral profile of the light source in the absence of Rb vapor, *I* is the spectral profile of the light after passing through the sample of path length *l*, and σ is the absorption cross section specific to a given electronic Rb transition. The Rb density can then be calculated as

(12) $$\left[\mathrm{Rb}\right]=\frac{1}{\pi r_{0}cfl}\int \ln\left(\frac{I_{0}\left(\nu \right)}{I(\nu )}\right)d\nu ,$$

where

(13) $$\int \sigma \left(\nu \right)d\nu =\pi r_{0}cf.$$

**Figure 3:**
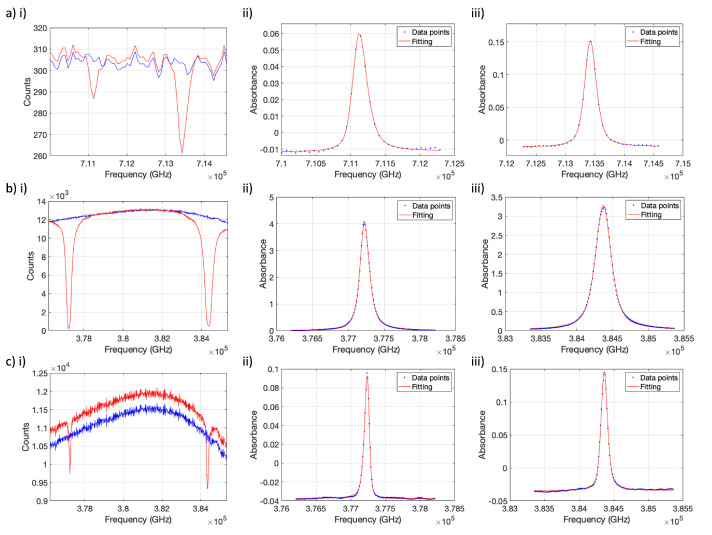
Example absorption spectra. (**a**) Violet and (**b**) NIR spectra acquired with the 1g Rb cell (Figure 1a), at cell position =+36 cm from the center of the cell, above the Rb source, at oven temperature, $T_{\mathrm{oven}}∼$20 °C, $I_{0}\left(\nu \right)$ (blue) and $T_{\mathrm{oven}}=145\;{}^{\circ}$C, I(ν) (red). Corresponding pseudo-Voigt fitted absorbance spectra for the $5S_{1/2}\to$ (**aii**) $6P_{1/2}$, (**aiii**) $6P_{3/2}$, (**bii**) $5P_{1/2}$ (D${}_{1}$), (**biii**) $5P_{3/2}$ (D${}_{2}$) transitions. Due to the high optical thickness for D${}_{1}$ and D${}_{2}$ absorption (deviation from the Beer-Lambert law), the lineshape becomes less well defined and the quality of the fit worsens. (**c**) Example of NIR spectra acquired at cell position =−36 cm from the center of the cell, away from the Rb source, at $T_{\mathrm{oven}}∼$20 °C, $I_{0}\left(\nu \right)$ (blue) and $T_{\mathrm{oven}}=125\;{}^{\circ}$C, where low [Rb] is observed (the Beer-Lambert law is obeyed). The offset between the cold and hot NIR spectra is due to variation when the optical fiber is frequently moved between spectrometers and is accounted for in the baseline fitted parameter, *B*.

$r_{0}$ is the classical electron radius, *c* is the speed of light and *f* is the absorption oscillator strength specific to each Rb transition, as given in Table 2. Pseudo-Voigt lineshape fitting is applied to absorbance spectra $S\left(\nu \right)$ for each transition, taking the form

(14) $$S\left(\nu \right)+B=\ln\left(\frac{I_{0}\left(\nu \right)}{I(\nu )}\right)+B=A\left[\eta L\left(\nu \right)+\left(1-\eta \right)G\left(\nu \right)\right]+B,$$

where η is the relation coefficient between $L\left(\nu \right)$ and $G\left(\nu \right)$, *B* is the baseline correction and *A* is the baseline corrected integral of $S\left(\nu \right)$ [49], as shown in Figure 3. $L\left(\nu \right)$ is the normalized Lorentzian,

(15) $$L\left(\nu \right)=\frac{\Delta \nu /2\pi }{{\left(\nu -\nu_{0}\right)}^{2}+{\left(\frac{\Delta \nu }{2}\right)}^{2}},$$

and $G\left(\nu \right)$ is the normalized Gaussian,

(16) $$G\left(\nu \right)=\frac{2}{\Delta \nu }\sqrt{\frac{\ln2}{\pi }}\exp\left[-4\ln2{\left(\frac{\left(\nu -\nu_{0}\right)}{\Delta \nu }\right)}^{2}\right],$$

where $\nu_{0}$ and Δν are the temperature/pressure-dependent center frequency and linewidth, respectively [47]. Lineshape asymmetry is accounted for by defining Δν as

(17) $$\Delta \nu \left(\nu \right)=\frac{2\Delta \nu_{0}}{1+\exp\left[a\left(\nu -\nu_{0}\right)\right]},$$

where *a* is the asymmetry parameter and $\Delta \nu_{0}$ is the symmetric FWHM (i.e., when a=0) [49]. Differences in ${}^{85}$Rb and ${}^{87}$Rb absorption was not considered as $\nu_{0}$ differences are smaller than Δν (order 100 GHz) for both near-IR (D${}_{1}$ and D${}_{2}$, $|\nu_{0}^{{}^{85}\mathrm{Rb}}-\nu_{0}^{{}^{87}\mathrm{Rb}}|∼$8GHz [50,51]) and violet (5${}^{2}$S${}_{1/2}\to 6^{2}P_{1/2}$, $|\nu_{0}^{{}^{85}\mathrm{Rb}}-\nu_{0}^{{}^{87}\mathrm{Rb}}|=110$ MHz and 5${}^{2}$S${}_{1/2}\to 6^{2}P_{3/2}$, $|\nu_{0}^{{}^{85}\mathrm{Rb}}-\nu_{0}^{{}^{87}\mathrm{Rb}}|=130$ MHz [52]) transitions. The Rb vapor density can then be calculated using

(18) $$\left[\mathrm{Rb}\right]=\frac{A}{\pi r_{0}cfl}.$$

The curvature of the SEOP cell leads to small changes in the path length of the probe beam off-center of the cell. A fixed path length must be used for absorption spectroscopy as variation in the path length leads to systematic errors in the measured absorbance. Given that the geometry of the cell is fixed, and that the beam width must be balanced with the need for high signal, we evaluated the path length variability. The path length is defined as the mean path length calculated from the chord in a circle as

(19) $$\bar{l}=\frac{1}{r_{b}}\int_{0}^{r_{b}}2\sqrt{\left(r_{\mathrm{cell}}^{2}-x^{2}\right)}dx.$$

Carrying out the integral in Equation (19) gives

(20) $$\bar{l}=\frac{r_{\mathrm{cell}}^{2}}{r_{b}}\arcsin\left(\frac{r_{b}}{r_{\mathrm{cell}}}\right)+\sqrt{\left(r_{\mathrm{cell}}^{2}-r_{b}^{2}\right)},$$

where $r_{b}$ is the probe beam radius and $r_{\mathrm{cell}}$ is the internal SEOP cell radius, as given in Table 2. The minimum path length sampled is only 4% shorter than the maximum path length, and the mean path length is 1.4% shorter than the maximum path length.

### 2.4. Acquisition Procedure

A total acquisition time of 2 min was used for the violet spectrometer and 1 min for the NIR spectrometer. The integration time was adjusted to closely match the dynamic range of the spectrometer, whilst being careful to avoid signal saturation. This was typically 20 ms with 6000 scan averages for violet acquisitions and 4 ms with 15,000 scan averages for NIR spectra. Background spectra were also taken on the same day as cold and hot cell spectra, with the bulb switched off. These were subtracted from hot and cold spectra to reduce systematic uncertainties in absorbance spectra.

Absorption spectroscopy spectra were acquired with the optical pumping laser switched off to avoid any possible emission due to energy pooling [54], as observed in NIR absorption spectroscopy of Rb during Rb-${}^{129}$Xe SEOP by Kelley and Branca [20]. With the pump laser off, laser heating would no longer be present, leading to changes in [Rb] over time. To minimize [Rb] distribution changes, the cell was closed during absorption spectroscopy acquisitions. This also limited the total acquisition time, limiting scan averages. Absorption spectra with an SNR<8 were not included due to >20% mean absolute percentage error determined from fitting known synthetic spectra, as detailed in Appendix A.

[Rb] measurements were performed on a closed cell in the absence of the optical pumping laser, for a range of oven temperatures, $T_{\mathrm{oven}}$, in order to identify the range of accurate [Rb] measurement for each absorption line. The cell was filled with 3% enriched Xe (86% ${}^{129}\mathrm{Xe}$), 10%N${}_{2}$, balanced with He to 1.47 bar at 20 °C, which is equivalent to the same number density as 2 bar at 125 °C (nominal running conditions). The cell containing 1g Rb in the main body of the cell was used, and absorption spectroscopy was performed with the probe beam positioned at the back of the cell, 36 cm from the center, directly above the Rb source.

For [Rb] measurements at different cell positions during continuous-flow SEOP, a flow rate of 2000 sccm (standard cubic cm per minute for standard conditions T=20 °C and p=1 atm) at 2 bar was used. A 180 W pumping laser at 794.77 nm was used (QPC Lasers Inc BrightLock Ultra-500). The SEOP cell was opened to allow gas flow and the power meter reading and cell temperatures, $T_{\mathrm{cell}}$, from separate thermocouples adhered to the top of the external cell surface, were recorded. After 3 min, a small amount of gas was dispensed to measure the $P_{\mathrm{Xe}}$. The cell was then closed, the pump laser was powered off and violet ($5S_{1/2}\to$$6P_{1/2}$ and $5S_{1/2}\to$$6P_{3/2}$) absorption spectra were recorded. Immediately afterwards, the optical fiber was swapped from the violet spectrometer to the NIR spectrometer to record D${}_{1}$ and D${}_{2}$ absorption spectra.

### 2.5. ${}^{129}$Xe Polarimetry and Laser Absorption

${}^{129}$Xe polarization was measured by dispensing the flowing gas into a separate pyrex cylinder. This was then placed inside a solenoid NMR coil placed within the polarizer $B_{0}$ field and an FID was acquired. The signal was then compared to a ${}^{1}$H reference signal acquired at the same frequency (32.8 kHz), within an identical cylinder filled with CuSO${}_{4}$ doped water. See Appendix B for further details on ${}^{129}$Xe polarimetry. Laser power absorption measurements were also performed by measuring the power at the back of the SEOP cell during continuous-flow SEOP and whilst at 20 ${}^{\circ }$C (i.e [Rb]=0).

### 2.6. Spin-Exchange Cross Section

The ability to measure [Rb] directly should improve the accuracy and confidence of $\gamma^{\prime }$ and $\Gamma^{\prime }$ determined from $\Gamma_{\mathrm{up}}$ or $\Gamma_{\mathrm{down}}$ measurements ($\Gamma_{\mathrm{down}}=\gamma_{\left(P_{\mathrm{Rb}}=0\right)}^{\prime }\left[\mathrm{Rb}\right]+\Gamma^{\prime }$). While $\Gamma^{\prime }$ is primarily governed by cell wall relaxation, and can therefore determine the condition of the cell wall in terms of polarizer performance, $\gamma^{\prime }$ should be a constant for fixed running conditions, and can be compared to the theoretical framework derived from the literature outlined in this paper. The $P_{\mathrm{Rb}}$ dependence of $\gamma_{\mathrm{vdW}}^{\prime }$ leads to differing $\gamma^{\prime }$ whether $\Gamma_{\mathrm{up}}$ or $\Gamma_{\mathrm{down}}$ measurements are performed. $\Gamma_{\mathrm{down}}$ is measured for $P_{\mathrm{Rb}}=0$ conditions, whilst $\Gamma_{\mathrm{up}}$ is measured for $P_{\mathrm{Rb}}>0$. $\Gamma_{\mathrm{up}}$ measurements require the pumping laser to be on, affecting simultaneous absorption spectroscopy, and so were not performed.

At each $T_{\mathrm{oven}}$, the center of the SEOP cell was probed with absorption spectroscopy, directly below the NMR coil, to measure [Rb]. Once sufficient ${}^{129}$Xe polarization build up had occurred, the pump laser diode was switched off and in-cell NMR acquisitions were taken periodically (TR = 30 to 90 s, 11 pulses/point with the exception of lowest [Rb] where 21 pulses were used) during ${}^{129}$Xe relaxation. Where the acquisition was sufficiently long, mean [Rb] values were calculated from measurements taken at the start and end of each acquisition. Once complete, initial amplitudes, including correction for T${}_{2}^{*}$ relaxation during the pulse-acquire delay, were fitted to exponential decay in order to calculate $\Gamma_{\mathrm{down}}$.

## 3. Results and Discussion

### 3.1. Closed Cell Rb Density for Different Oven Temperatures

**Figure 4:**
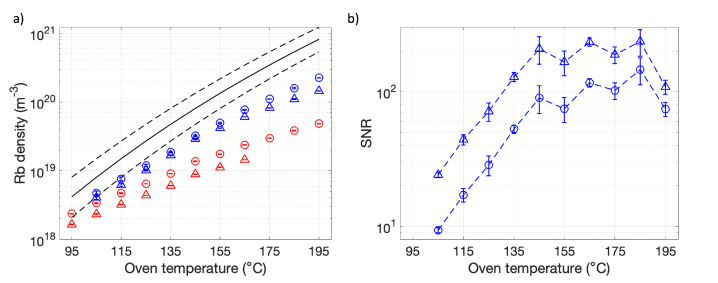
(**a**) Rb densities measured from the $5S_{1/2}\to$$6P_{1/2}$ (blue circles), $6P_{3/2}$ (blue triangles), $5P_{1/2}$ (D${}_{1}$) (red circles), $5P_{3/2}$ (D${}_{2}$) (red triangles) transitions vs. oven temperature, $T_{\mathrm{oven}}$. The black line is saturation Rb density, ${\left[\mathrm{Rb}\right]}_{\mathrm{sat}}$, calculated based on $T_{\mathrm{oven}}$ (see Table 1) and dashed lines indicate $T_{\mathrm{oven}}\pm 10\;{}^{\circ}$C. (**b**) Corresponding signal-to-noise ratio (SNR) for violet absorbance spectrum. SNR in D${}_{1}$ and D${}_{2}$ absorbance spectra was high (>100) and so was not included. Dashed blue lines are to guide the eye only.

[Rb] measurements to identify the range of accurate [Rb] measurement are shown in Figure 4. Three measurements were made at each $T_{\mathrm{oven}}$.

**Figure A2:**
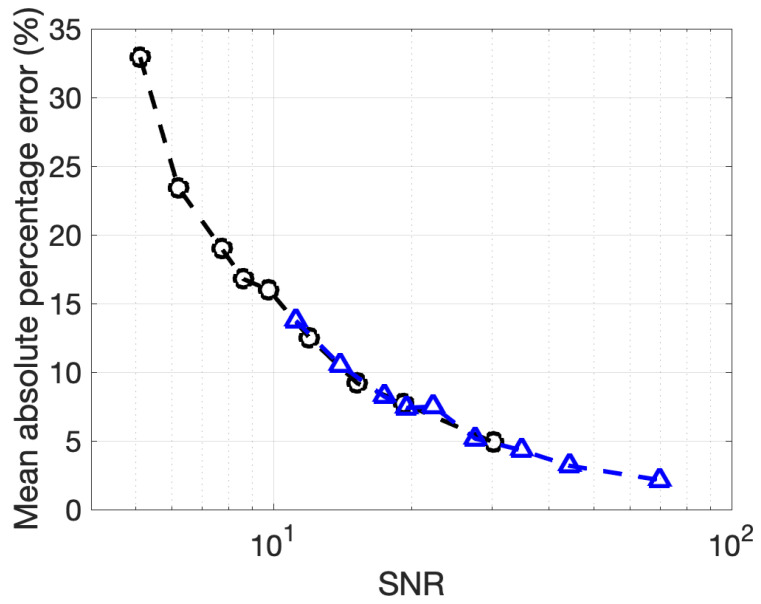
Mean absolute percentage error, *M*, vs SNR for $5S_{1/2}\to$$6P_{1/2}$ (black circles), $6P_{3/2}$, (blue triangles) for n=200 repeats. Dashed lines are to guide the eye only.

By comparing [Rb] for a given transition to [Rb] calculated from the closest and higher absorption oscillator strength transition, we quantify [Rb] measurement accuracy over a range of $T_{\mathrm{oven}}$. A discrepancy suggests a breakdown in the Beer-Lambert law whereas agreement suggests accurate [Rb] measurement. We define the Beer-Lambert law breakdown, our upper detection limit for accurate [Rb] measurement, as a 20% difference between [Rb] measurements for each transition, following the accuracy limits defined in the absorption spectroscopy lineshape fitting described in Appendix A (Figure A2). Figure 4a shows that [Rb] calculated from the D${}_{1}$ and D${}_{2}$ lines were consistently >28% lower than those predicted by the $6P_{1/2}$ and D${}_{1}$ absorption lines, respectively. This suggests a breakdown in the Beer-Lambert law for the D${}_{1}$ and D${}_{2}$ lines, leading to under prediction of [Rb] for these transitions over the range of temperatures evaluated. Incidentally, an upper accuracy limit for ${\left[\mathrm{Rb}\right]}_{D_{2}}$, and correspondingly imposed on ${\left[\mathrm{Rb}\right]}_{D_{1}}$, was determined in Section 3.3. At $T_{\mathrm{oven}}=155\;{}^{\circ}C$, ${\left[\mathrm{Rb}\right]}_{6P_{3/2}}=\left(4.16\pm 0.01\right)\times 10^{19}$ m${}^{-3}$ calculated from $6P_{3/2}$ absorption was found to be 16% lower than calculated from $6P_{1/2}$ absorption, suggesting deviation from the Beer-Lambert law, and under prediction of [Rb], for the $6P_{3/2}$ line at higher [Rb].

The lowest measurable [Rb] was ${\left[\mathrm{Rb}\right]}_{6P_{3/2}}=\left(4.1\pm 0.1\right)\times 10^{-18}$ m${}^{-3}$, which is 84.8% lower than ${\left[\mathrm{Rb}\right]}_{\mathrm{sat}}=2.70\times 10^{19}$ m${}^{-3}$ as defined in Table 1, for $T_{\mathrm{oven}}=125\;{}^{\circ}$C. This provides an order of magnitude range of sensitivity for ${\left[\mathrm{Rb}\right]}_{6P_{3/2}}$ measurements and was expected to be sufficient in order to assess [Rb] heterogeneity in our large SEOP cells.

Figure 4 shows experimentally measured [Rb] lower than ${\left[\mathrm{Rb}\right]}_{\mathrm{sat}}$ for most $T_{\mathrm{oven}}$ investigated. This is similar to Shao et al. [41] and Shang et al. [55] who both observed lower than saturation Rb vapor densities over a range of temperatures. Adsorption of alkali metal to glass walls has been suggested as a mechanism for lower alkali metal vapor densities [56]. In addition, the large volume of the SEOP cell in our setup likely leads to long [Rb] build up times, highlighting the need to use a sufficiently long presaturator column to reach full Rb saturation at high gas flow rates through the SEOP cell [46].

### 3.2. Absorption Spectroscopy during Continuous-Flow SEOP

**Figure 5:**
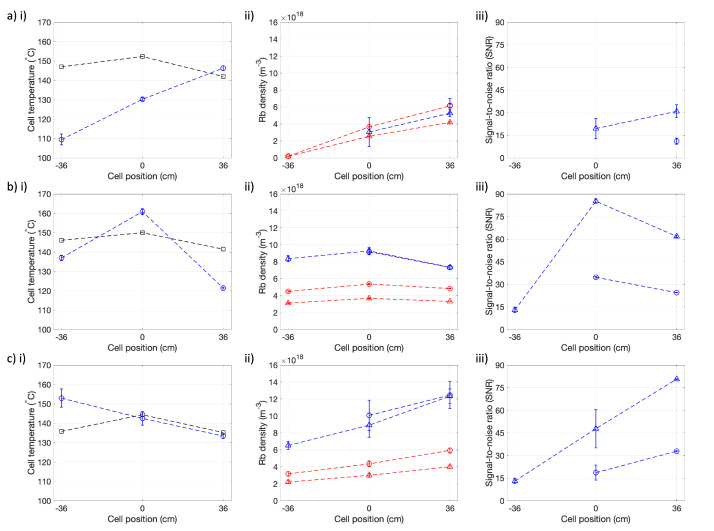
(**i**) Cell temperature, $T_{\mathrm{cell}}$, (**ii**) Rb density, [Rb] (**iii**) signal-to-noise ratio (SNR) in violet absorbance spectra at different cell positions for (**a**) 1g Rb main body cell, (**b**) 5 g Rb main body cell and (**c**) 2 g Rb presaturator cell (see Figure 1). $T_{\mathrm{cell}}$ was measured during continuous flow SEOP (blue circles) and with the cell closed without the pumping laser present (black squares). [Rb] measured from the $5S_{1/2}\to$$6P_{1/2}$ (blue circles), $6P_{3/2}$ (blue triangles), $5P_{1/2}$ (D${}_{1}$) (red circles), $5P_{3/2}$ (D${}_{2}$) (red triangles) transitions. Dashed lines are to guide the eye only. The presaturator temperature was measured to be 164.0±0.8 °C. SNR in D${}_{1}$ and D${}_{2}$ absorbance spectra was high (>100) and so was not included in (**iii**) plots. N.B.: in (**ai**), (**bi**) and (**ci**) $T_{\mathrm{cell}}$ measured without the pumping laser present do not have an error bars due to only one measurement recorded.

[Rb] measurements during continuous-flow SEOP were performed at different cell positions for 3 different Rb source distributions. Measurements were repeated three times for each cell position probed. [Rb] and $T_{\mathrm{cell}}$ distributions are presented in Figure 5, and $P_{\mathrm{Xe}}$ and optical pumping laser power absorbed are presented in Table 3.

In order to decouple laser heating and oven performance in cell temperature heterogeneity measurements, stable cell temperatures were recorded with the optical pumping laser switched off, as shown in Figure 5.

Absorption spectroscopy measurements from an initial flow-through were typically treated as anomalous due to high variation in recorded measurements. The initial flow-through was considered necessary in order to disturb [Rb] and better match closed cell and under-flow thermal conditions to improve thermal stability. Even with this improvement in thermal stability, SEOP cell and oven temperatures, as well as laser power absorbed, fluctuated over long time scales (order 10 min) whilst the SEOP cell was closed. For the presaturator cell, fluctuations resulted in every other repeat acquisition showing lower absorption (see Table 3) and corresponding lower [Rb] at each cell position probed. This may be a result of reduced coupling of the Rb source in the presaturator from the oven ambient air temperature compared to SEOP cells with Rb sources in the main body of the cell, leading to non-replenishment of [Rb] in the main body of the cell once the cell has cooled and is reheated during closed cell conditions. However, further investigation into decoupling [Rb] from laser heating and the oven temperature controller in order to control [Rb] over a wider range of conditions is needed, as well as optimisation of closed cell [Rb] in between continuous-flow cycles.

Each run through of flowing gas through the cell was started when $T_{\mathrm{oven}}=125\;{}^{\circ}$C and the transmitted power was increasing (power absorbed decreasing), suggesting decreasing [Rb], as a decreasing transmitted power may suggest Rb runaway conditions, which are unstable and difficult to reproduce.

Figure 5a shows that the 1g Rb cell under flow conditions produces maximum [Rb] at the back of the cell with ${\left[\mathrm{Rb}\right]}_{6P_{1/2}}=\left(6.2\pm 0.2\right)\times 10^{18}$ m${}^{-3}$. This is 77% lower than ${\left[\mathrm{Rb}\right]}_{\mathrm{sat}}$ for $T_{\mathrm{oven}}=125\;{}^{\circ}$C. [Rb] decreases towards the front of the cell. The 9.3% lower ${\left[\mathrm{Rb}\right]}_{D_{2}}$ than ${\left[\mathrm{Rb}\right]}_{D_{1}}$ at cell position =−36 cm compared to 31.8% at cell position =+36 cm suggests improved accuracy in ${\left[\mathrm{Rb}\right]}_{D_{1}}$. This value suggests a 96.6% reduction in [Rb] from the back to the front of the cell. $T_{\mathrm{cell}}$ also follows this distribution with $T_{\mathrm{cell}}$ hottest at the back of the cell, $T_{\mathrm{cell}}=146\pm 1\;{}^{\circ}$C, and decreasing towards the front of the cell, $T_{\mathrm{cell}}=110\pm 3\;{}^{\circ}$C. This is due to the Rb source being located at the back of the cell, leading to a local build up in [Rb] at this position. Under flow, we would expect this [Rb] to move towards the front of the cell. However, the initial high [Rb] at the back of the cell leads to local heating in this region. As the oven can only control the global ambient air temperature within the oven, the effective heating from the oven reduces. This most likely results in lower heating of the cell in the region of the cell where laser heating is lower, which in this case is towards the front of the cell where there is no Rb source and [Rb] is low, leading to $T_{\mathrm{cell}}$ heterogeneity. This means that as gas with an initial high [Rb] flows from the back to the front of the cell, Rb will be deposited on the cell walls due to the lower $T_{\mathrm{cell}}$, resulting in an unwanted reduction in [Rb]. This unexpectedly low [Rb] resulted in few violet transition points with a SNR >8 that could be processed.

Figure 5b shows that the 5 g Rb cell produces maximum [Rb] at the center of the cell with ${\left[\mathrm{Rb}\right]}_{6P_{3/2}}=\left(9.3\pm 0.4\right)\times 10^{18}$ m${}^{-3}$. In addition, [Rb] heterogeneity is significantly lower for the 5 g Rb cell than the 1 g Rb cell, with a 21.5% difference in the maximum to lowest [Rb] (${\left[\mathrm{Rb}\right]}_{6P_{3/2}}=\left(7.3\pm 0.2\right)\times 10^{18}$ m${}^{-3}$). This is due to the higher surface area of the Rb source that extends towards the center of the SEOP cell, allowing for greater Rb evaporation and build up in [Rb] in the main body of the SEOP cell. In addition, the greater laser heating towards the front of the cell due to the location of Rb sources in this area results in higher $T_{\mathrm{cell}}$, which reduces Rb condensation and maintains high [Rb] in this region. It is worth noting, however, that the 5 g Rb cell would often end up in Rb runaway, where total laser absorption would occur and the front of the SEOP cell would reach high $T_{\mathrm{cell}}$ (>200 °C). This would most likely leave the majority of the cell unilluminated, resulting in low volume-averaged $P_{\mathrm{Rb}}$ and therefore low $P_{\mathrm{Xe}}$ of Xe gas exiting the SEOP cell.

Figure 5c shows that the 2 g Rb presaturator cell produces maximum [Rb] at the back of the cell equal to ${\left[\mathrm{Rb}\right]}_{6P_{1/2}}=\left(1.2\pm 0.2\right)\times 10^{19}$ m${}^{-3}$. This is 56% lower than ${\left[\mathrm{Rb}\right]}_{\mathrm{sat}}$ for $T_{\mathrm{oven}}=125\;{}^{\circ}$C. [Rb] decreases towards the front of the cell, similar to the 1 g Rb cell, where at cell position =−36 cm, ${\left[\mathrm{Rb}\right]}_{6P_{3/2}}=\left(6.5\pm 0.5\right)\times 10^{18}$ m${}^{-3}$, which is 46% lower than [Rb] measured at cell position =+36 cm. Therefore, [Rb] homogeneity in the 5 g Rb presaturator cell is greater than in the 1g Rb cell, but less than in the 5 g Rb cell.

The 5 g Rb cell and 2 g Rb presaturator produced higher $P_{\mathrm{Xe}}$ than the 1g Rb cell, likely due to the more homogeneous and higher [Rb], increasing the spin-exchange rate and leading to a greater build up in $P_{\mathrm{Xe}}$. Measured $P_{\mathrm{Xe}}$ values are ∼ factor-2 lower than those measured from previous work with the same 1g Rb cell design [15]. This may be due to the higher gas pressure of 2 bar used in this work compared to 1.25 bar in previous work [15]. We would expect a 21–33% lower $P_{\mathrm{Xe}}$ when operating at 2 bar compared to 1.25 bar, for $1/\Gamma^{\prime }=44$ min to 80 s, based on the modelling framework outlined in ref. [15].

Furthermore, $T_{\mathrm{cell}}$ measurements in the absence of the pumping laser revealed systematic $T_{\mathrm{cell}}$ differences to $T_{\mathrm{oven}}$. $T_{\mathrm{cell}}$ measurements were not reported in our previous study [15], making comparison of oven performance difficult. If we assume that the $T_{\mathrm{cell}}$ offset to $T_{\mathrm{oven}}$ was not present in ref. [15], then $T_{\mathrm{oven}}=125\;{}^{\circ}$C in this work is likely far from the optimal to provide "peak" $P_{\mathrm{Xe}}$, and as such $P_{\mathrm{Xe}}$ is significantly lower.

A limitation of the oven is the single thermocouple used for global temperature control of the oven. For the work in Figure 5, the thermocouple was placed approximately halfway between the center and the back of the oven, and for the work in Figure 4, the oven thermocouple was placed approximately halfway between the center and the front of the oven. This is to be as far as possible from the oven heated air inlets which are located at the front, center and back of the oven, where ambient oven temperature heterogeneity is suspected to be greatest and temperature stability to be the most challenging. However, this is likely to bias temperature control, both of the oven and the cell, to the thermocouple region.

Additional limitations of this work are that measurements were only taken at 3 different cell positions and $P_{\mathrm{Xe}}$ measurements did not consider ${}^{129}$Xe depolarization due to dark Rb in outlet tubes. This depolarization is likely to be more significant with higher [Rb] in the front of the cell and also dependent on Rb deposition in the outlet tubes.

Experiments using the 1g Rb cell were performed 2 to 6 months after the cell was installed on the polarizer, in this time thermal cycling and other experiments, involving sparse polarizer use, were performed. The 5 g Rb cell and the 2 g Rb presaturator cell were installed and ∼3 days of thermal cycling was performed before experiments were carried out. Thermal cycling involves running the polarizer at high and varying temperatures in order to evaporate and deposit Rb on the cell walls. The cells were then checked to see if reproducible laser power absorption, suggestive of stable [Rb], could be achieved before experiments were carried out. The cause of [Rb] changes and reduced $P_{\mathrm{Xe}}$ with extensive cell use are currently not well defined in the literature and require further investigation.

### 3.3. $\gamma^{\prime }$ and $\Gamma^{\prime }$ Measurements

**Figure 6:**
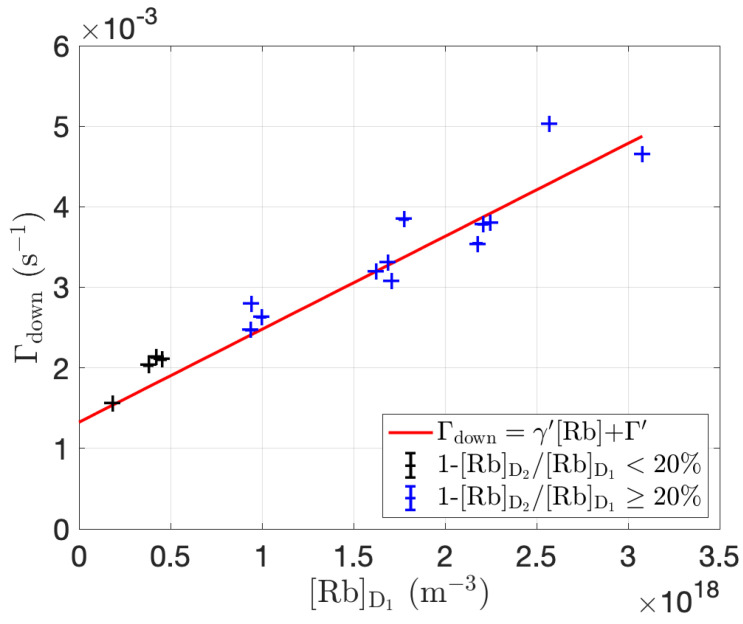
${}^{129}$Xe relaxation rate, $\Gamma_{\mathrm{down}}$, as a function of [Rb] for 1 g Rb main body cell. [Rb] measured from D${}_{1}$ line absorption. $\gamma^{\prime }=1.2\pm 0.1\times 10^{-21}$ m${}^{3}$s${}^{-1}$ and $1/\Gamma^{\prime }=13\pm 2$ min. >20% difference in ${\left[\mathrm{Rb}\right]}_{D_{1}}$ and ${\left[\mathrm{Rb}\right]}_{D_{2}}$, suggestive of deviation from the Beer-Lambert law for D${}_{2}$ absorption, occurs at ${\left[\mathrm{Rb}\right]}_{D_{2}}≳4\times 10^{17}$ m${}^{-3}$ and correspondingly ${\left[\mathrm{Rb}\right]}_{D_{1}}≳5\times 10^{17}$ m${}^{-3}$, which is our current limit of accurate ${\left[\mathrm{Rb}\right]}_{D_{1}}$ measurement.

Using the 1g Rb cell, $\Gamma_{\mathrm{down}}$ was measured at different oven temperatures ($T_{\mathrm{oven}}=80\;{}^{\circ}$C to 125 °C). [Rb] was measured simultaneously using atomic absorption spectroscopy. $\Gamma_{\mathrm{down}}$ was plotted against [Rb], and $\gamma^{\prime }$ and $\Gamma^{\prime }$ were determined from a linear fit, as shown in Figure 6.

**Figure 7:**
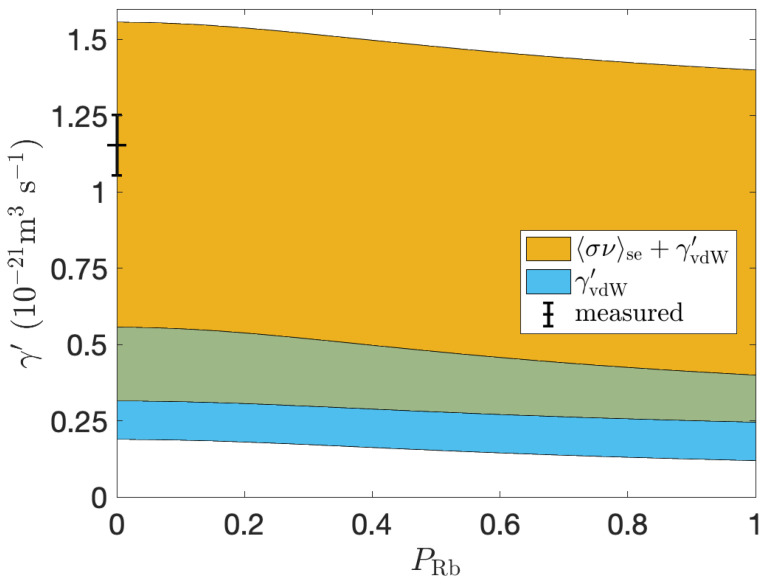
Theoretical Rb-${}^{129}$Xe spin-exchange cross section, $\gamma^{\prime }$, as a function of Rb polarization, $P_{\mathrm{Rb}}$, as calculated for our conditions (p = 2 bar, T = 125 ${}^{\circ }$C and 3%Xe, 10%N${}_{2}$, 87%He). The blue region is the range of contribution due to three-body van der Waals interactions, and the yellow region is the added range of contribution due to measured binary spin-exchange cross sections from the literature. The green region is where the derived ranges of each spin-exchange cross section contribution from the literature overlap. Our measured value is plotted at $P_{\mathrm{Rb}}=0$, as the optical pumping laser was not on during $\Gamma_{\mathrm{down}}$ measurements.

Theoretical $\gamma^{\prime }$ was also calculated, using values in Table 1, and plotted as a function of $P_{\mathrm{Rb}}$, as shown in Figure 7. Ranges of theoretical binary and vdW spin-exchange contributions were included in this plot to reflect the variation in the literature.

Figure 7 shows that $\gamma^{\prime }$ is within the range of predicted spin-exchange rates. Our measurement is likely an overestimation due to >20% difference in ${\left[\mathrm{Rb}\right]}_{D_{1}}$ and ${\left[\mathrm{Rb}\right]}_{D_{2}}$, suggestive of deviation from the Beer-Lambert law for D${}_{2}$ absorption, which occurs at ${\left[\mathrm{Rb}\right]}_{D_{2}}≳4\times 10^{17}$ m${}^{-3}$ and correspondingly ${\left[\mathrm{Rb}\right]}_{D_{1}}≳5\times 10^{17}$ m${}^{-3}$. This is our current limit of accurate ${\left[\mathrm{Rb}\right]}_{D_{1}}$ measurement, as an upper accuracy limit for ${\left[\mathrm{Rb}\right]}_{D_{1}}$ cannot be determined due to SNR < 8 in violet absorption spectra. Thus we expect the true spin-exchange rate value to be lower. Figure 7 also shows the $P_{\mathrm{Rb}}$ dependence for our conditions, thus we expect a 10% to 22% decrease in the spin-exchange cross section during optical pumping conditions. It is also shown that for $P_{\mathrm{Rb}}=0$, there is an up to 5 times difference in the total spin-exchange cross section depending on which values from the literature are used, suggesting further investigation into spin-exchange parameters is needed.

We note that when ${\left[\mathrm{Rb}\right]}_{\mathrm{sat}}$ calculated from $T_{\mathrm{oven}}$ were used instead of those measured using absorption spectroscopy, $\gamma^{\prime }=\left(7\pm 4\right)\times 10^{-23}$ m${}^{3}$s${}^{-1}$ and $1/\Gamma^{\prime }=\left(8\pm 4\right)$ min. This highlights the issues with measuring $\gamma^{\prime }$ and $\Gamma^{\prime }$ assuming saturation Rb densities in systems where actual [Rb] differs significantly. Given that temperature dependence of wall relaxation is not currently well defined in the literature, a limitation with this method of measuring $\gamma^{\prime }$ and $\Gamma^{\prime }$ is that it assumes cell wall relaxation is temperature and/or [Rb] independent, which may not be a valid assumption as reported in studies of both ${}^{3}$He-Rb [57] and ${}^{129}$Xe-Rb [58] SEOP.

## 4. Future Work

Higher and higher laser powers with narrow linewidths are being used on modern spin polarizer systems, which makes managing laser heating considerations increasingly important. Further efforts to understand and improve thermal management on our spin polarizer system is therefore necessary to maximize the potential for future enhancements of this technology. We have demonstrated an improvement in [Rb] homogeneity, however greater control of [Rb] in large SEOP cell setups through improved understanding of the gas flow and cell thermodynamics, and thermal management considerations warrants further investigation.

For hyperpolarized ${}^{129}$Xe production rates to be improved, determining optimal temperature conditions for use with the Rb presaturator cell is necessary. SNR improvement, by using a dedicated LED or laser diode violet light source, in violet absorption spectroscopy will extend the lower limit of detectable [Rb], improving accuracy of future $\gamma^{\prime }$ and $\Gamma^{\prime }$ measurements.

## 5. Conclusions

We have evaluated the accuracy limits, due to deviation in the Beer-Lambert law and low SNR considerations, of absorption spectroscopy in measuring [Rb] within a high throughput ${}^{129}$Xe-Rb polarizer over a range of running conditions. Violet Rb electronic transitions were found to be valid for calculating [Rb] of the order $<5\times 10^{19}$ m${}^{-3}$, whereas the D${}_{1}$ and D${}_{2}$ NIR transitions were found to underpredict [Rb] for [Rb] of the order $>5\times 10^{17}$ m${}^{-3}$. [Rb] heterogeneity was found to be greatest within a cell containing a localized drop of 1g Rb. [Rb] homogeneity was improved within the cell containing a line of 5 g Rb covering half the total cell length, as well as within a cell containing a 2 g presaturator region. Runaway conditions observed in the 5 g Rb cell however indicate using a presaturator cell is likely the most favourable Rb source distribution.

While $\gamma^{\prime }$ was measured and shown to be in line with current theory from the literature, it is worth noting the large range of published values of constants used to estimate both binary and molecular spin exchange rates. Further optimisation to improve [Rb] homogeneity and thermal management, as well as improving the accuracy of [Rb] measurement by increasing SNR in violet absorption spectroscopy, is needed to improve the accuracy of future spin-exchange rate measurements on large-cell SEOP systems.

## Figures and Tables

**Table 1:** SEOP Parameters. A range of values is given where multiple, differing, applicable values are present in the literature.

Parameter	Description	Equation/Value	Reference
${\left[G\right]}_{0,\mathrm{Xe}}$	Xe characteristic gas density	$\left(\frac{28.3\mathrm{Torr}}{760\mathrm{Torr}}\right)\;\cdot \;\left(\frac{273.15K}{349K}\right)\;\cdot \;{\left(\frac{349K}{T}\right)}^{1/2}$ amg	[34,35,36]
${\left[G\right]}_{0,N_{2}}$	N${}_{2}$ characteristic gas density	$\left(\frac{103\mathrm{Torr}}{760\mathrm{Torr}}\right)\;\cdot \;\left(\frac{273.15K}{349K}\right)\;\cdot \;{\left(\frac{349K}{T}\right)}^{1/2}$ amg	[35], with *T* dependence from [34]
${\left[G\right]}_{0,\mathrm{He}}$	He characteristic gas density	$\left(\frac{175\mathrm{Torr}}{760\mathrm{Torr}}\right)\;\cdot \;\left(\frac{273.15K}{358.45K}\right)\;\cdot \;{\left(\frac{358.45K}{T}\right)}^{1/2}$ amg	[37], with *T* dependence from [34]
$\eta_{85}$	Relative abundance of ${}^{85}$Rb	0.7215	-
$\eta_{87}$	Relative abundance of ${}^{87}$Rb	0.2785	-
${\langle \sigma \nu \rangle }_{\mathrm{se}}$	Binary ${}^{129}$Xe-Rb spin-exchange cross section	$\left(1.26-10\right)\times 10^{-22}$ m${}^{3}$s${}^{-1}$ (specific values: $\left(1.26,4.02,4.1,10\right)\times 10^{-22}$ m${}^{3}$s${}^{-1}$)	[38], [39], [35,40], [41] respectively
*k*	Molecular chemical equilibrium constant	244 ${\text{Å}}^{3}$ ${\left(T/373\right)}^{-3/2}$	[33]
$\frac{\omega }{2\pi }$	Spin-rotation frequency of the Rb electron spin vector **S** about the rotational angular momentum vector **N** of the RbXe molecule	γN/h=109−170MHz (specific values: (109, 121, 130, 140−170) MHz)	[42], [33,43], [43], [36] respectively
*x*	The Breit-Rabi field parameter	3.2, 4.1	[35], [44] respectively
${\left[\mathrm{Rb}\right]}_{\mathrm{sat}}$	Saturation Rb vapor density	$\frac{10^{10.55-\frac{4132K}{T}}}{k_{B}T}\times 10^{-1}$ m${}^{-3}$	[22]

**Table 2:** Absorption spectroscopy parameters.

Parameter	Description	Value	Reference
$f_{D_{1}}$	Absorption oscillator strength for $5S_{1/2}\to 5P_{1/2}$ (D${}_{1}$)	0.3422	[50,51]
$f_{D_{2}}$	Absorption oscillator strength for $5S_{1/2}\to 5P_{3/2}$ (D${}_{2}$)	0.6957	[50,51]
$f_{6P_{1/2}}$	Absorption oscillator strength for $5S_{1/2}\to 6P_{1/2}$	$3.87\times 10^{-3}$	[53]
$f_{6P_{3/2}}$	Absorption oscillator strength for $5S_{1/2}\to 6P_{3/2}$	$9.46\times 10^{-3}$	[53]
$r_{\mathrm{cell}}$	Internal cell radius	37.5 mm	This work
$r_{b}$	Probe light beam radius	10.55 mm	This work
*l*	Path length, defined by Equation (20)	74 mm	This work

**Table 3:** Xe polarization, $P_{\mathrm{Xe}}$, and optical pumping laser power absorbed for each Rb SEOP cell tested.

Rb Distribution	$P_{\mathrm{Xe}}$ (%)	Power Absorbed (W)
1 g Rb main body	15.3±0.7	46±2
5 g Rb main body	17.9±0.4	108±1
2 g Rb presaturator	17.6±0.7	121±2
2 g Rb presaturator ^1^	15.8±1.1	100±2

^1^ Presaturator cell fluctuations resulted in every other acquisition showing lower optical pumping laser power absorption.

**Figure A1:**
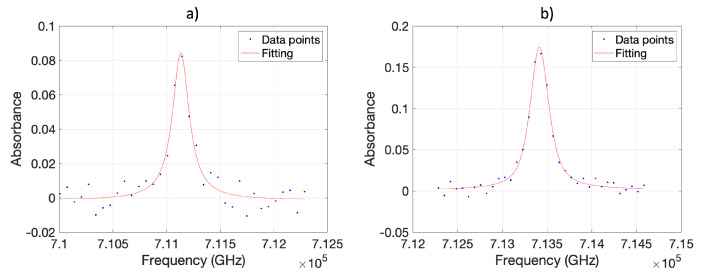
Example synthetic absorption spectra. (**a**) Voigt fitting parameters $A_{F}=25.43$, $a_{F}=-3.62\times 10^{-5}$ GHz${}^{-1}$, $B_{F}=-0.00133$, $\Delta \nu_{0,F}=189.27$ GHz, $\eta_{F}=0.999$, $\nu_{0,F}=711,131$ GHz, M=15.59%, SNR=11.78 for the $5S_{1/2}\to$$6P_{1/2}$ transition. (**b**) Voigt fitting parameters $A_{F}=62.77$, $a_{F}=-2.98\times 10^{-4}$ GHz${}^{-1}$, $B_{F}=-0.00133$$\Delta \nu_{0,F}=250.87$ GHz, $\eta_{F}=0.814$, $\nu_{0,F}=713,414$ GHz, M=10.12%, SNR=23.66 for the $5S_{1/2}\to$$6P_{3/2}$ transition.

**Figure A3:**
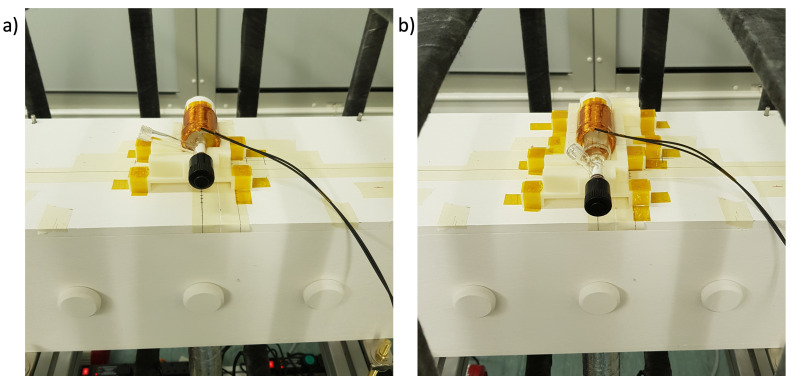
${}^{129}$Xe polarimetry setups with each container containing Xe gas mixture inside a solenoid coil, placed on the 3D printed stand(s). (**a**) Spherical container setup, (**b**) Cylindrical container setup.

**Figure A4:**
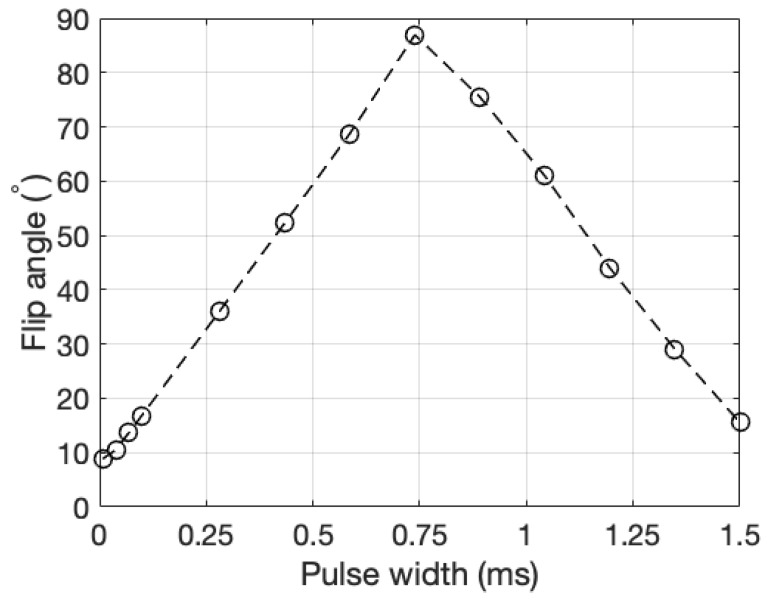
${}^{129}$Xe flip angle measurements for an amplitude of 2.4 V, at 32.8 kHz. Dashed lines are to guide the eye only.

**Figure A5:**
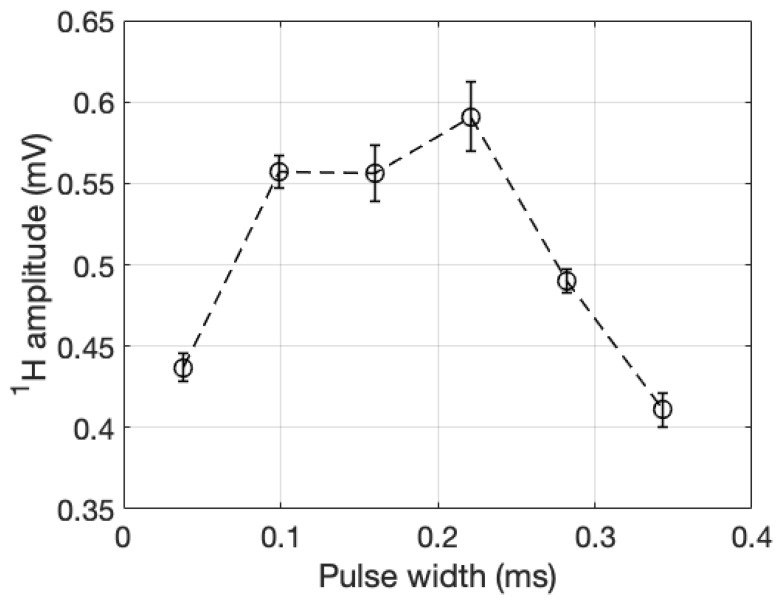
${}^{1}$H amplitude vs pulse width for a pulse amplitude of 2.4 V, at 32.8 kHz. Dashed lines are to guide the eye only.

**Figure A6:**
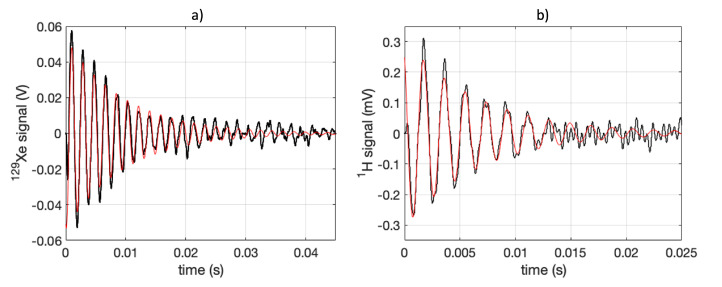
FIDs acquired on the polarizer. The black line is the signal and the red line is the fitted FID. (**a**) 1 scan ${}^{129}$Xe FID, with V=53.6 mV and $T_{2}^{*}=9.76$ ms, using a pulse width of 0.89 ms (**b**) 20,000 scan average ${}^{1}$H FID, with V=0.307 mV and $T_{2}^{*}=6.71$ ms, using a pulse width of 0.22 ms.

**Figure A7:**
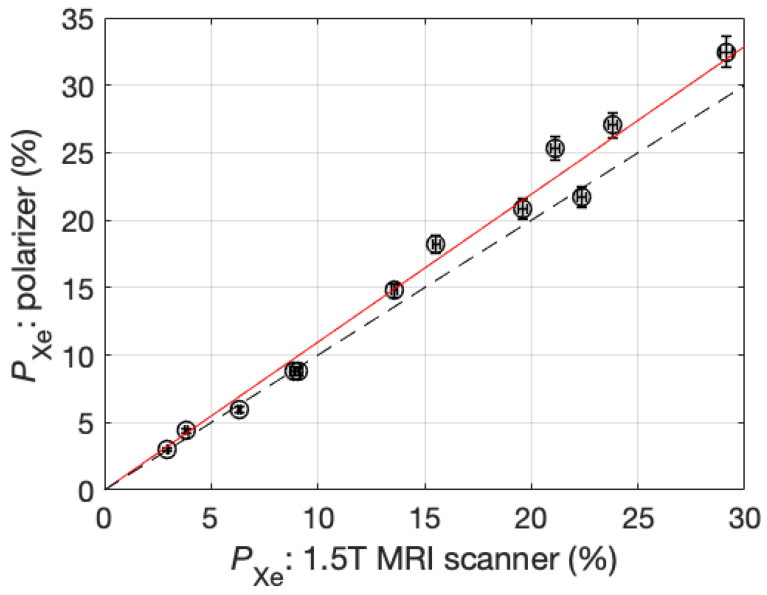
${}^{129}$Xe polarization measured on the polarizer ($P_{\mathrm{Xe}}^{pol}$) vs. on the 1.5 T scanner ($P_{\mathrm{Xe}}^{sc}$). $P_{\mathrm{Xe}}^{pol}=m*P_{\mathrm{Xe}}^{sc}$ was fitted where m=1.10±0.05, $R^{2}=0.9825$ (red). The dashed line indicates $P_{\mathrm{Xe}}^{pol}=P_{\mathrm{Xe}}^{sc}$, highlighting the gradient offset that may be the result of depolarization between the polarizer and the 1.5T MRI scanner.

## Data Availability

Not applicable.

## Appendix A. Accuracy of Absorption Spectroscopy Fitting

In order to assess the accuracy of the absorption spectroscopy fitting routine, synthetic violet absorption spectroscopy spectra were produced. Voigt profiles were produced using Equation (14), with parameters $A_{S}=22$, $a_{S}=-0.001$ GHz${}^{-1}$, $B_{S}=0$, $\Delta \nu_{0,S}=212$ GHz, $\eta_{S}=0.7$, $\nu_{0,S}=711,134$ GHz for the $5S_{1/2}\to$$6P_{1/2}$ transition and $A_{S}=57$, $a_{S}=0$ GHz${}^{-1}$, $B_{S}=0$, $\Delta \nu_{0,S}=237$ GHz, $\eta_{S}=0.7$, $\nu_{0,S}=713,414$ GHz for the $5S_{1/2}\to$$6P_{3/2}$ transition. The frequencies used were the same as the spectrometer in order to match the resolution of the synthetic violet spectra to the measured violet spectra. Gaussian noise was then added to the exponential of the Voigt profiles using MATLAB (MathWorks) function "awgn". The resulting synthetic spectra are then produced by taking the natural log of the result, as shown in Figure A1.

Different amounts of gaussian noise were added in order to vary the signal-to-noise ratio (SNR) in each *i* absorbance spectra, and *n* repeats performed. Pseudo-voigt fitting was then applied to calculate the integral, $A_{F,i}$. The mean absolute percentage error was then calculated as

(A1) $$M=\frac{100\%}{n}\sum_{i=1}^{n}\left|\frac{A_{S,i}-A_{F,i}}{A_{S,i}}\right|,$$

where $A_{S,i}$ is the actual integral of the absorption line. *M* for different SNR values is shown in Figure A2.

From Figure A2, we threshold our measurements, where possible in this work, for an SNR of 8, corresponding to a mean absolute percentage error ∼20%.

## Appendix B. 129 Xe Polarimetry

### Appendix B.1. 129 Xe Polarimetry on 1.5T MRI Scanner Limitations

Within our group, ${}^{129}$Xe polarization is typically calculated by measuring ${}^{129}$Xe samples dispensed from a polarizer in our 1.5T GE MRI scanner. The signal is then compared to a thermal ${}^{129}$Xe sample acquired on the same setup. This has the advantage of reflecting the ${}^{129}$Xe polarization we would expect when performing in vivo scans on this scanner. However, potential polarization losses between dispensing and acquisition on the scanner may occur. In addition, scanner time is often limited, as well as being expensive due to scanner running costs.

In-cell polarization measurements could be performed, however ${}^{129}$Xe number densities may be difficult to determine as gas temperatures are likely different to outer cell surface temperatures. Also, sampling the entire cell with homogeneous B${}_{1}$ would require complex RF engineering. In addition, ${}^{129}$Xe polarization heterogeneity across the cell is likely high, making sampling a smaller region less useful. Sampling the front of the SEOP cell would be more suitable, however, laser heating and heterogeneous temperatures would alter the coil resistance, which would complicate correction for precise ${}^{129}$Xe polarimetry. We therefore implemented a ${}^{129}$Xe polarimetry system external to the SEOP cell but within the SEOP B${}_{0}$ field for ease of dispensing ${}^{129}$Xe gas and measuring its polarization.

### Appendix B.2. Coil, Stand and Sampling Container

A 200 turn solenoid coil (CnC Tech Magnet Wire MW28-C SL AWG 26) with dimensions 47.0 mm long and formed around a 34.6 mm diameter PVC tube was constructed. This coil design was chosen as solenoids produce a well defined B${}_{1}$ homogeneous region, unlike a surface coil, allowing flip angles to be measured via RF destruction, as discussed later. At 32.8 kHz, the coil Q was determined using an S${}_{11}$ measurement to be ∼42. A 3D printed stand was made to fix the position of the solenoid coil and sampling container within the B${}_{0}$ field, as shown in Figure A3. The center of the oven lid was determined to be the most homogeneous region of the B${}_{0}$ coil outside the oven and as such the most appropriate place to position the solenoid coil and stand. The solenoid configuration is oriented perpendicular to the optical pumping cell to ensure ${}^{129}$Xe spins in the optical pumping cell are not sampled. The oven lid becomes warm during polarizer operation, so the stand has feet which allow air flow around the stand, ensuring the solenoid and sample temperatures do not significantly change, whilst the room air conditioning and fans maintain air room temperature.

Two sampling containers were used. A spherical (diameter = 23.9 mm) container was initially used which was positioned within the homogeneous region of the solenoid. However, low signal to noise with 3%Xe gas mixture and ${}^{1}$H signals, even with thermal signal averaging, meant a larger cylindrical container was built, with a diameter and length of 28.1 cm and 95.5 mm respectively. Both sampling containers were made of pyrex and cleaned before use. No anti-relaxation surface coatings were applied. The $T_{1}$ relaxation time constant was measured to be 56±5 min for the spherical container and 79±2 min for the cylindrical container. The $T_{1}$ of the cylinder is likely longer than the spherical container $T_{1}$ due to the lower surface to volume ratio, as well as the spherical container being cleaned ∼1 year earlier than than the cylinder when the $T_{1}$ measurements were performed. However, $T_{1}$ of the spherical container is still sufficiently long for flip-angle measurements using short repetition times to be performed. A Teflon stopcock and chemthread on each container ensured leak-free connections to the gas manifold. The containers can then be filled, disconnected from the gas manifold and placed in the solenoid on the stand where a free induction decay (FID) is taken.

Radiation damping, if present, can lead to distortion in hyperpolarized ${}^{129}$Xe FIDs, decreasing accuracy of Xe polarimetry. Radiation damping was investigated by observing T${}_{2}^{*}$ changes with successive pulses for different flip angles. The spherical container on a separate clinical polarizer with higher B${}_{0}$ homogeneity, and as such longer T${}_{2}^{*}$ was used. 100% enriched Xe samples collected from cyrogenic separation were used. No significant change in T2* was observed for flip angles up to $90^{\circ }$. Given the small number of pulses used (typically 1 $90^{\circ }$ pulse) and short T${}_{2}^{*}$, radiation damping effects did not have a noticeable effect on any of the recorded hyperpolarized ${}^{129}$Xe FIDs.

Flip-angle calibration measurements were performed with 100% enriched Xe samples, in order to maximize NMR sensitivity when using low flip angles. Subsequent acquisitions with a $90^{\circ }$ flip angle used lean gas mixture (3% enriched Xe) samples to reduce time and gas mixture usage. 100% enriched Xe samples were collected whilst under-flow (Q = 2000 sccm) in the cryo-trap, during a 2 min freeze out. Lean Xe gas samples were dispensed directly from the SEOP cell into an evacuated tedlar bag, and then into the evacuated sampling container. The tedlar bag allows the sampling container pressure to be maintained at 1 atm during filling, without depolarizing which can be caused by pressure regulator valves. Once the sampling container is filled, it is sealed, disconnected from the gas manifold and placed on the Xe polarimetry stand, inside the solenoid coil. A twin container was filled with ∼10 mM of CuSO${}_{4}$ to reduce the T${}_{1}$ to allow thermal averaging with 400 ms repetition times (TR) for ${}^{1}$H acquisitions [59]. ${}^{1}$H acquisitions with TR=1 s and 400 ms were found to be consistent, so TR=400 ms is sufficiently long to perform thermal averaging.

### Appendix B.3. NMR Spectrometer

A home-built NMR spectrometer is used for acquisitions [60]. Some modifications were implemented in order to improve signal strength for ${}^{1}$H acquisitions, namely (i) an updated DAQ card with a 2MHz sampling rate was used (National Instruments NI 6361) and (ii) a pre-amplifier (Standford Research Systems SR560 Low-noise Voltage Preamplifier) and duplexer, as developed by Antonacci et al. [29]. The amplifier was set to a gain of 1000. Uncertainty in amplifier gain linearity is mitigated by using the same amplifier gain for ${}^{129}$Xe and ${}^{1}$H acquisitions. The dynamic range of the DAQ was set to match the expected signal, avoiding saturating the DAQ and reducing noise. The amplifier gain is limited by the maximum output signal of the amplifier and the dynamic range of the DAQ. Timing of the acquisition process is performed via the internal trigger of the DAQ card. Parnell et al. [60] timed the transmit pulse and receive channel to begin successively and confirmed the synchronisation. We included the pre-amplifier blanking pulse in this synchronisation. Preamp blanking delay times up to 16 ms were achieved and 4 ms was typically used, which is sufficiently long for the transmit pulse width and coil ring down to occur in our setup with the acquisition parameters used.

### Appendix B.4. Flip Angle Calibration

${}^{129}$Xe flip angles, $\alpha_{{}^{129}\mathrm{Xe}}$, were measured with multiple pulses with TR=1s ($\mathrm{TR}<<T_{1}$), so that RF destruction is the sole contribution to the decrease in signal between pulses, and fitted to $\log V_{{}^{129}\mathrm{Xe}}=\left(n-1\right)\;\cdot \;\ln\cos\alpha_{{}^{129}\mathrm{Xe}}$, where $V_{{}^{129}\mathrm{Xe}}$ is the initial signal amplitude found using FID fitting and *n* is the pulse number. Due to the heterogeneous B${}_{1}$ across the sample when using the cylindrical sampling container, flip angle measurements using RF destruction could not be performed directly on this container, however flip angle calibration on the spherical container should hold for the different containers. For a fixed pulse amplitude of 2.4 V, a pulse width of 74 ms produced a flip angle closest to $90^{\circ }$, as shown in Figure A4. A pulse width of 89 ms, which gives a flip angle of $75.5^{\circ }$, was mistakenly used for measurements. As such, a correction factor of $\sin90^{\circ }/\sin75.5^{\circ }=1.0329$ was multiplied to ${}^{129}$Xe polarization values.

${}^{1}$H flip angle calibration was performed by taking pulse averaging scans for different pulse lengths. Initial amplitudes were then calculated from FID fitting, and then a correction factor applied to account for T${}_{2}^{*}$ relaxation during the pre-acqusition delay. Due to the low SNR of ${}^{1}$H acquisitions, the cylindrical container was used for these measurements. During ${}^{1}$H flip angle measurements, the number of pulses was scaled by $\frac{20,000}{\sqrt{\sin\left(\alpha_{{}^{1}H}/90^{\circ }\right)}}$, where $\alpha_{{}^{1}H}$ is the expected flip angle to maintain the same SNR. For a fixed pulse amplitude of 2.4 V, a pulse width of 22 ms produced the highest amplitude signal, corresponding to $\alpha_{{}^{1}H}=90^{\circ }$, as shown in Figure A5.

### Appendix B.5. Xe Polarization Calculation

The Xe polarization is calculated as

(A2) $$P_{{}^{129}\mathrm{Xe}}=P_{{}^{1}H}\;\cdot \;\frac{V_{{}^{129}\mathrm{Xe}}}{V_{{}^{1}H}}\;\cdot \;\frac{\sin\alpha_{{}^{1}H}}{\sin\alpha_{{}^{129}\mathrm{Xe}}}\;\cdot \;\frac{I_{{}^{1}H}}{I_{{}^{129}\mathrm{Xe}}}\;\cdot \;\frac{\left[{}^{1}H\right]}{\left[{}^{129}\mathrm{Xe}\right]}\;\cdot \;\frac{\gamma_{{}^{1}H}}{\gamma_{{}^{129}\mathrm{Xe}}}\;\cdot \;C_{T_{2}^{*}},$$

where $P_{{}^{1}H}$ is the thermal polarization of sampled ${}^{1}H$ nuclei, which is temperature and B${}_{0}$ field dependent. $\left[{}^{1}H\right]$ and $\left[{}^{129}\mathrm{Xe}\right]$ are the nuclei number densities. *I*, γ and *V* are the nuclear spin, gyromagnetic ratio and initial signal amplitude for each nucleus. *V* and $T_{2}^{*}$ are determined by FID fitting, as shown in Figure A6. Differences in pulse length and $T_{2}^{*}$ are accounted for as $C_{T_{2}^{*}}=\exp\left[{\left(\frac{T_{AQ}}{T_{2}^{*}}\right)}_{{}^{129}\mathrm{Xe}}-{\left(\frac{T_{AQ}}{T_{2}^{*}}\right)}_{{}^{1}H}\right]$. For the purposes of our spectrometer, the pulse-acquire delay, $T_{AQ}=T_{CO}-\tau$, where τ is the pulse width and $T_{CO}=5$ ms is the cutoff time. The dominant source of uncertainty in $P_{{}^{129}\mathrm{Xe}}$ is the uncertainty in $V_{{}^{1}H}$.

### Appendix B.6. Confirmation with 1.5T MRI Scanner

In order to confirm the accuracy of the Xe polarization values measured on the polarizer, Xe polarization was simultaneously measured on the 1.5T MRI scanner. Figure A7 shows fair agreement between Xe polarization calculated by both methods over a range of ${}^{129}$Xe polarizations. The higher Xe polarizations measured on the polarizer compared to the 1.5T MRI scanner may be due to depolarization between the polarizer and the 1.5T MRI scanner.
